# Environmental Enrichment During Adolescence Mitigates Cognitive Deficits and Alcohol Vulnerability due to Continuous and Intermittent Perinatal Alcohol Exposure in Adult Rats

**DOI:** 10.3389/fnbeh.2020.583122

**Published:** 2020-09-25

**Authors:** Anna Brancato, Valentina Castelli, Gianluca Lavanco, Carla Cannizzaro

**Affiliations:** ^1^Department of Health Promotion, Mother and Child Care, Internal Medicine and Medical Specialties of Excellence “G. D’Alessandro”, University of Palermo, Palermo, Italy; ^2^Department of Biomedicine, Neuroscience and Advanced Diagnostics, University of Palermo, Palermo, Italy; ^3^INSERM U1215, NeuroCentre Magendie, Bordeaux, France; ^4^University of Bordeaux, Bordeaux, France; ^5^Department of Biomedical and Biotechnological Sciences, Section of Pharmacology, University of Catania, Catania, Italy

**Keywords:** alcohol, perinatal binge alcohol drinking, perinatal continuous alcohol drinking, declarative memory, spatial memory, alcohol vulnerability, environmental enrichment

## Abstract

Perinatal alcohol exposure affects ontogenic neurodevelopment, causing physical and functional long-term abnormalities with limited treatment options. This study investigated long-term consequences of continuous and intermittent maternal alcohol drinking on behavioral readouts of cognitive function and alcohol vulnerability in the offspring. The effects of environmental enrichment (EE) during adolescence were also evaluated. Female rats underwent continuous alcohol drinking (CAD)—or intermittent alcohol drinking paradigm (IAD), along pregestation, gestation, and lactation periods—equivalent to the whole gestational period in humans. Male offspring were reared in standard conditions or EE until adulthood and were then assessed for declarative memory in the novel object recognition test; spatial learning, cognitive flexibility, and reference memory in the Morris water maze (MWM); alcohol consumption and relapse by a two-bottle choice paradigm. Our data show that perinatal CAD decreased locomotor activity, exploratory behavior, and declarative memory with respect to controls, whereas perinatal IAD displayed impaired declarative memory and spatial learning and memory. Moreover, both perinatal alcohol-exposed offspring showed higher vulnerability to alcohol consummatory behavior than controls, albeit perinatal IAD rats showed a greater alcohol consumption and relapse behavior with respect to perinatal-CAD progeny. EE ameliorated declarative memory in perinatal CAD, while it mitigated spatial learning and reference memory impairment in perinatal-IAD progeny. In addition, EE decreased vulnerability to alcohol in both control and perinatal alcohol-exposed rats. Maternal alcohol consumption produces drinking pattern-related long-term consequences on cognition and vulnerability to alcohol in the offspring. However, increased positive environmental stimuli during adolescence may curtail the detrimental effects of developmental alcohol exposure.

## Introduction

Perinatal exposure to alcohol can affect *in utero* neurodevelopment, causing both physical and functional long-term alterations (Dejong et al., 2019). Despite pre-conceptional alcohol cessation is recommended, alcohol drinking during pregnancy is prevalent worldwide, especially in Europe (Popova et al., 2017). One of the best predictors of alcohol use throughout the perinatal period is the pattern of alcohol use before pregnancy; indeed, women who report binge or heavy drinking prior to pregnancy likely maintain it during pregnancy and throughout lactation (Davidson et al., 1981; Ethen et al., 2009; Mallard et al., 2013; Anderson et al., 2014; Kitsantas et al., 2014), increasing the risk for growth deficits, facial dysmorphology, and behavioral and neurocognitive abnormalities in the progeny (Viljoen et al., 2005; May et al., 2007; Urban et al., 2008). Aside from the more severe fetal alcohol syndrome (FAS), “fetal alcohol spectrum disorders” (FASD) have been recently characterized as a broad range of deficits observed in the child when exposed to alcohol at any time prenatally (Dejong et al., 2019). Those alterations involve memory, attention, affective and social behavior, abnormal responses to stress and natural rewards (American Psychiatric Association, 2013), and susceptibility to drug and alcohol abuse later in life (Baer et al., 2003; Alati et al., 2006; Glantz and Chambers, 2006).

While the consequences related to heavy prenatal alcohol exposure are generally acknowledged, the assessment of the neurobehavioral alterations potentially produces by low-to-moderate alcohol exposure in humans displays mixed results (Kelly et al., 2013; Flak et al., 2014; Kilburn et al., 2015). This may be due to a number of methodological issues—most of the studies focus on physical malformations—and confounding variables, such as the unreliable self-reports about the degree of alcohol exposure (number of drinks per week rather than amount at one session) and the underestimation of subtle neurobehavioral deficits which may appear later in life (Conover and Jones, 2012).

Preclinical models of maternal alcohol drinking can enhance our understanding of the adverse outcomes secondary to developmental alcohol exposure. Indeed, fetal alcohol exposure in humans can be modeled by perinatal alcohol exposure in rats, since the full gestational period in rodents is equivalent to the first and second trimesters in humans, while the first 10 postnatal days in rats correspond to the third trimester in humans (Patten et al., 2014). Besides, high levels of alcohol consumption can be induced in Sardinian alcohol-preferring and Wistar female rats by manipulating the schedule of alcohol access (Loi et al., 2014; Brancato et al., 2016). First developed and characterized in male rats (Wise, 1973; Simms et al., 2008), the intermittent access procedure in the two-bottle choice paradigm, consisting of cycles of drinking and abstinence, leads to a rapid increase in voluntary alcohol consumption, in comparison with continuous access to alcohol (Carnicella et al., 2014). Rats exposed to this procedure consume the most abundant amount of their daily total intake within the first hour of availability of the alcohol bottle, reaching intoxicating blood alcohol levels in a short period of time (about mg/dl after the first 30 min–1 h, Simms et al., 2008; Carnicella et al., 2009; Loi et al., 2014). This procedure models a voluntary binge-like drinking pattern (Crabbe et al., 2011; Sprow and Thiele, 2012; Sabino et al., 2013; Carnicella et al., 2014; Spear, 2018; Jeanblanc et al., 2019) and, as such, may represent a valuable tool to model drinking trajectories during pregnancy and lactation. Interestingly, when female rats are exposed to a long-term binge-like intermittent alcohol drinking (IAD) paradigm, they display a significant decrease in alcohol consumption during pregnancy and resume excessive alcohol consumption during the lactation period (Brancato et al., 2016).

Thus, in the present study, we aimed at investigating whether the binge-like IAD paradigm, resulting in higher and irregular peaks of blood alcohol levels in the dams, could lead to distinct long-term consequences on cognition and vulnerability to alcohol abuse in the offspring, with respect to continuous alcohol drinking (CAD), which produces steady lower peaks of blood alcohol levels, even in the face of overall high levels of exposure. On the other hand, even the exposure to low to moderate blood alcohol concentrations can cause significant neuronal damage, when it occurs during the neurodevelopmental window such as throughout gestation (Patten et al., 2014). Therefore, it follows that according to time, dosage, and duration of perinatal alcohol exposure, different developmental alterations thus, may occur.

The long-term cognitive effects of perinatal alcohol exposure, either continuous or intermittent, were assessed in the adult offspring, through a multidimensional behavioral battery, including declarative memory in the novel object recognition test, spatial learning, cognitive flexibility, and reference memory in the Morris water maze (MWM). Vulnerability to excessive alcohol drinking, in terms of rate of voluntary alcohol consumption and relapse behavior after a period of forced abstinence, was assessed using a two-bottle “alcohol vs. water” choice drinking paradigm.

It is worth noting that treatment strategies to prevent or mitigate perinatal alcohol-related deficits are currently very limited (Murawski et al., 2015). In this regard, growing evidence supports a beneficial role of the exposure to positive stimuli during sensitive time windows of brain development. Indeed, the environmental-enrichment (EE), experimental paradigm consisting of housing conditions that include novelty, social interaction and exercise, enhances sensory, cognitive, and motor stimulation, which, in turn, translates into increased neuroplasticity in brain regions critical for emotional regulation, cognitive functions and reward sensitivity (Bayat et al., 2015; Crofton et al., 2015; Morera-Herreras et al., 2019). However, conflicting evidence is reported when the effect of EE was evaluated toward motivational effects of drugs of abuse, including alcohol (Nithianantharajah and Hannan, 2006; Solinas et al., 2009; Pautassi et al., 2017; Rae et al., 2018). Thus, while it is critical to identify maternal alcohol consumption as a primary target to prevent fetal consequences, we investigated whether EE during adolescence could prevent or mitigate the effects of perinatal alcohol exposure on behavioral readouts of cognitive function and alcohol vulnerability.

## Materials and Methods

### Animals, Perinatal Alcohol Exposure, and Rearing Conditions

The methods used for perinatal alcohol exposure and breeding have been previously reported in detail (Brancato et al., 2018).

Briefly, adult female Wistar rats (200–220 g, Envigo, Italy) were housed individually in standard rat cages (40 × 60 cm, 20 cm in height), with *ad libitum* access to water and food, in a temperature- (22 ± 2°C) and humidity- (55 ± 5%) controlled room, on a 12-h light/dark cycle (08:00–20:00).

Rats were gently handled for 3 min per day for a week before the experimental procedures, when they were randomly assigned to one of the three experimental groups, according to the two-bottle choice self-administration paradigm: water drinking controls (CTRL), CAD, and IAD. Female rats underwent the self-administration procedure during pre-gestation (12 weeks), gestation (3 weeks), and post-gestation (3 weeks) periods, accordingly to the respective home-cage two-bottle “alcohol vs. water”-choice-drinking paradigm.

Indeed, CTRL rats were given two bottles of tap water. CAD rats were given a 24-h free choice between one bottle of alcohol (20% v/v) and one of tap water, 7 days per week; IAD rats were given 24-h alcohol (20% v/v) access during 3 days per week, i.e., on Monday, Wednesday, and Friday, while they received two bottles of tap water on the intervening days.

Plastic bottles (120 ml; metal cap 0.8-mm-diameter hole, Tecniplast, Italy) were filled every day with 100 ml of 20% alcohol (daily prepared from alcohol 96° (Carlo Erba Reagents, Italy) diluted with tap water) and presented at lights-off in an alternative left–right position in order, to avoid side preference. Rats were weighed daily, and alcohol and water intake was measured 1 h after lights-off and the day after, immediately before lights-off, by weighing the bottles. Possible fluid spillage was monitored by using multiple bottles filled with water and alcohol 20%, allocated in empty cages interspersed in the racks (Loi et al., 2014).

At the end of the 12-week two-bottle choice drinking paradigm, each female rat was housed with a single breeder. The day when pregnancy was confirmed by vaginal smear (Cannizzaro et al., 2008; Plescia et al., 2014b), designed as gestational day 1 (GD1), eight female rats were randomly selected from each experimental group (*n* = 12), housed in standard maternity cages, filled with wood shavings. Dams were inspected twice daily for delivery until the day of parturition, considered as postnatal day 0 (PND 0); dams and litters were kept in a nursery (24 ± 2°C) and not separated until weaning, in order to model the human condition and avoid confounding factors (Subramanian, 1992; Wilson et al., 1996; Santangeli et al., 2016). Mean alcohol consumption at 1 h and 24 h by CAD and IAD rat dams during pre-conception period, gestation, and lactation was recorded and reported as g/kg ± SEM. After weaning, two male rats from each litter of the three drinking groups were randomly assigned to either the standard (SE) or enriched (EE) rearing environment, so that the experimental groups of rat offspring were perinatal water-exposed controls (p-CTRL SE, *n* = 8); perinatal continuous alcohol-exposed rats (p-CAD SE, *n* = 8); perinatal intermittent alcohol-exposed rats (p-IAD SE, *n* = 8); perinatal water-exposed controls + EE (p-CTRL EE, *n* = 8); perinatal continuous alcohol-exposed rats + EE (p-CAD EE, *n* = 8); and perinatal intermittent alcohol-exposed rats + EE (p-IAD EE, *n* = 8). In detail, from PND 21 onward, the rats reared in SE conditions were housed in pair in standard rat cages and left undisturbed by the experimenters except for weekly cage change, whereas the EE rats were group-housed (8/cage) in large cages (60 × 45 × 76 cm) with pet toys, pots, hideouts, ropes, running wheel, ladder, tunnel and plastic boxes, etc., which were relocated or changed daily to create novelty (Griva et al., 2017).

Experiments were approved by the Committee for the Protection and Use of Animals of the University of Palermo, in accordance with the current Italian legislation on animal experimentation (D.L. 26/2014) and the European directive (2010/63/EU) on care and use of laboratory animals. Every effort was made to minimize the number of animals used and their sorrow.

### Behavioral Procedures

The offspring were tested for behavioral reactivity in the open-field test at PND 55, for declarative memory in the novel object recognition test at PND 56–58, and for spatial learning, memory, and cognitive flexibility in MWM from PND 60 to PND 65. Afterward, they were assessed for alcohol vulnerability, in terms of rate of voluntary alcohol consumption in the induction and relapse-like phases of the two-bottle choice drinking paradigm, from PND 66 to PND 143.

The experiments were carried out in a sound-isolated room between 9:00 and 14:00. On the test days, rats were acclimatized to the testing room for 60 min before the experimental session. Rats’ performance was recorded and monitored in an adjacent room. The equipment was thoroughly cleaned in between each test, to avoid that rats’ behavior was affected by the detection of other rats’ scent.

### Open-Field Test

Behavioral reactivity in a novel environment was tested in the open-field test. The open-field arena is a Plexiglas square box (44 × 44 × 44 cm where locomotor activity and explorative behavior were measured) by employing an automatic video-tracking system (AnyMaze, Ugo Basile, Italy), in a mean light- (100 lx) illuminated chamber. Each experimental session lasted 5 min (Cannizzaro et al., 2016). The video-tracking system produces a quali-quantitative mapping of the motor pattern, measuring total distance traveled (TDT, m), as a measure of locomotor activity in a novel environment.

### Novel Object Recognition Test

Declarative learning and memory were tested in the novel object recognition test, as previously described (Brancato et al., 2020). On day 1, a 5-min habituation session was performed at 10.00 a.m., in order to let the animals freely explore the arena (44 × 44 × 44 cm) in a dim light-illuminated room. Twenty-four hours after the habituation session, rats underwent a 5-min training session when they were presented with two identical, nontoxic objects (i.e., two red metal cans) which were placed against a wall in the open-field arena. To prevent coercion to explore the objects, rats were released against the center of the opposite wall with its back to the objects. The time spent on exploring each object was recorded by using the AnyMaze video-tracking system (Stoelting Europe); a 2-cm^2^ area surrounding the objects was defined such that nose entries were recorded as time exploring the object. After the training session, animals were placed in their home cage for the retention interval. Then, animals were returned to the arena for the test session, 24 h after the training session. During the 5-min test session, the arena was equipped with two objects, one was identical to the one presented in the training session (i.e., familiar); the other was a novel object (a yellow hard plastic cup/a green hard plastic pepper). Objects were randomized and counterbalanced across animals. Objects and arena were thoroughly cleaned at the end of each experimental session. Time spent on exploring familiar and novel objects was recorded during both training and test sessions. The recognition index (RI%), i.e., the percentage of time spent on investigating the novel object, out of the total object investigation time [RI % = Time novel object/(Time novel object + Time familiar object)%], is a measure of novel object recognition and the main index of recognition memory. If RI% is higher than 50%, it indicates that the rat spent more time investigating the novel object, thus recalling the memory of the familiar one.

### Morris Water Maze

Spatial learning, cognitive flexibility, and reference memory were assessed in the MWM, by employing place learning, new place learning, and probe tasks (Cacace et al., 2011, 2012; Plescia et al., 2015) as described in detail below.

#### Apparatus

The MWM apparatus consisted of a circular, light-blue swimming pool with a diameter of 160 cm, and walls 70 cm high. It was filled with tap water to a depth of 50 cm. The water temperature was carefully maintained at 23 ± 2°C, and no agent was added to make the water opaque. The pool was divided into four quadrants of equal size by two imaginary diagonal lines running through the center, designated NW, NE, SW, and SE. A removable transparent escape platform (10 cm × 10 cm) was positioned in the middle of the quadrant, with the center 30 cm away from the wall and 1.5 cm below the water level, and not visible to the swimming rat. The pool was placed in an experimental room, decorated with several extra-maze cues (e.g., bookshelves and posters), and not modified throughout the entire experimental period. The experimental room was illuminated by a white light (60 W). The paths taken by the animals in the pool were monitored by a video camera mounted in the ceiling and recorded by the automatic video-tracking system (ANY MAZE, Ugo Basile, Italy).

#### Experimental Design

##### Place Learning (Days 1–3)

The Place learning task was employed to assess spatial learning and consisted of training the rats to escape from the water and reach the hidden platform placed in the SE zone, where it was maintained throughout the experimental session. The rat was introduced into the pool facing the wall of each quadrant, in the following order of starting points: NE, SW, NW, SE. Each rat underwent four trials a day, along 3 days, and was allowed to swim until the escape on the platform for a maximum of 90 s; escape latency was recorded as a measure of spatial learning and memory and reported as mean value of the four trials performed on each day of the experiment.

If the escape platform was reached, the rat was allowed to remain 15 s on it to reinforce the information on the visual–spatial cues in the environment. If the rat did not find the escape platform within 90 s, the experimenter guided gently the rat to the platform and allowed it to stay on it for 15 s. During the 5-min intertrial interval, rats were placed into their home cages and warmed under a heating lamp.

##### New Place Learning (Days 4–5)

The new place learning task was aimed at assessing rats’ cognitive flexibility. On the first day of task, the position of the escape platform was moved to the opposite quadrant (NW) compared to the place learning session. In this task, the rat was required to learn the new location of the platform during four trials, and escape latency was recorded as a measure of new spatial information acquisition, i.e., reversal learning. On the second day, the position of the platform was maintained in the same quadrant as in the first day of the new place learning task. The escape latency was recorded as a measure of acquisition and retrieval of the spatial information necessary to reach the platform location. Starting points, trial duration, inter-trial interval, reinforcement time on the platform, and any other experimental condition were the same as in the previous days.

##### Probe Test (Day 6)

Twenty-four hours after the last place learning session, rats were returned to the water maze for the probe test, aiming at assessing reference memory at the end of learning. The hidden platform was removed from the pool, and rats were allowed to swim freely for 90 s. The amount of time spent in the quadrant where the platform was previously located (target quadrant) was used as an index of the rat’s spatial reference memory.

### Two-Bottle “Alcohol vs. Water” Choice Drinking Paradigm

The offspring underwent the two-bottle “alcohol vs. water”-choice drinking paradigm (modified from Cacace et al., 2011) and were given 24-h free choice between one bottle of alcohol (10% v/v) and one of tap water, 7 days per week, for 8 weeks (induction period), followed by a 2-week relapse period, after 7 days of alcohol deprivation. 10% alcohol was daily prepared by diluting alcohol 96° (Carlo Erba Reagents, Italy) with tap water.

Plastic bottles (120 ml; metal cap 0.8 mm diameter hole, Tecniplast, Italy) were filled with 100 ml solution every day and presented at lights-off in an alternative left–right position, to avoid side preference. Alcohol and water intake were measured by weighing the bottles. Possible fluid spillage was monitored by using multiple bottles filled with water and 10% alcohol, positioned in empty cages interspersed in the cage racks (Loi et al., 2014). Rats’ body weight was daily monitored, and rats’ consummatory behavior was measured, in terms of g/kg of alcohol consumed along the drinking paradigm.

#### Statistical Analysis

Statistical analysis was performed using Prism 8, GraphPad Software, LLC, and IBM Statistical Package for the Social Sciences (SPSS) Statistics software (IBM, Armonk, NY, USA). Data were assessed for variance and normality by employing the Brown–Forsythe test and D’Agostino–Pearson omnibus K2 test, respectively, and for sphericity, by the Mauchly test. When data showed equal variance and normal distribution, the analysis included two-way analysis of variance (ANOVA), followed by Tukey’s multiple-comparison test to assess simple effects of the two different perinatal alcohol exposures, and repeated-measure ANOVA using the generalized linear model, with Bonferroni correction for pairwise comparisons. When data did not show normal distribution or sphericity, log-transformation and Geisser–Greenhouse correction were employed. Data are reported as mean ± SEM. Statistical significance was set at *p* < 0.05.

## Results

### Perinatal Alcohol Exposure and Developmental Data

Alcohol intake of CAD and IAD dams is reported in Table 1. Alcohol consumption did not affect maternal weight gain, litter size or pup birth weight, compared to controls.

**Table 1:** Mean alcohol consumption (g/kg) of continuous alcohol drinking (CAD) rats and intermittent alcohol drinking (IAD) rats at pre-conception, gestation, and lactation time.

	CAD		IAD	
Period	1 h	24 h	1 h	24 h
Pre-conception	0.8 ± 0.2	3.5 ± 0.1	3.4 ± 0.2	8.1 ± 0.3
Gestation	2.1 ± 0.2	3.4 ± 0.4	2.6 ± 0.3	5.4 ± 0.6
Lactation	3.1 ± 0.3	5.6 ± 0.6	3.6 ± 0.4	8.5 ± 0.4

*Data refer to mean ± SEM of *n* = 8 female rats along the alcohol drinking paradigm*.

### EE Prevents Alcohol-Induced Alteration in Behavioral Reactivity in p-CAD Offspring

**Figure 1:**
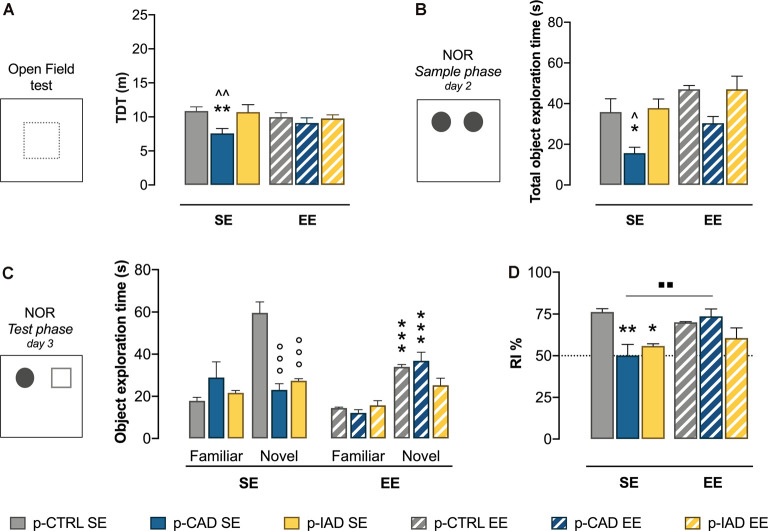
Effects of perinatal alcohol exposure and rearing conditions on locomotor activity and declarative memory. **(A)** In the open-field test, p-CAD SE offspring showed a significant decrease in locomotor activity (**p < 0.01 vs. p-CTRL SE, ^∧∧^*p* < 0.01 vs. p-IAD SE). **(B)** In the sample phase of the NOR test, p-CAD SE progeny showed a significant decrease in total object exploration (*p < 0.05 vs. p-CTRL SE; ^∧^*p* < 0.05 vs. p-IAD SE). **(C)** During the test phase of the NOR test, p-CAD SE and p-IAD SE rats displayed decreased exploration of the novel object, which was increased by environmental enrichment (EE) only in p-CTRL and p-CAD rats (°°°*p* > 0.001 vs. novel–p-CTRL SE; ***p > 0.001 vs. respective SE groups). **(D)** p-CAD SE and p-IAD SE rats showed decreased object discrimination in terms of recognition index. EE during adolescence was able to ameliorate the declarative memory performance in p-CAD offspring (*p < 0.05; **p < 0.01 vs. p-CTRL SE, ^▪▪^*p* < 0.01 vs. p-CAD SE). Each bar represents the mean ± SEM of *n* = 8 rats. p-CTRL, perinatal control; p-CAD, perinatal continuous alcohol drinking; p-IAD, perinatal intermittent alcohol drinking; SE, standard rearing environment; EE, enriched rearing environment. TDT, total distance travelled; NOR, novel object recognition.

Two-way ANOVA on log-transformed TDT data, including perinatal alcohol exposure and rearing conditions as statistical factors, highlights a significant main effect of perinatal alcohol exposure (*F*_(2,42)_ = 0.6.412, *p* = 0.01742). The Tukey multiple-comparison test indicates that p-CAD SE offspring showed a significant decrease in locomotor activity with respect to p-CTRL SE rats (*q* = 5.184, *df* = 42, *p* = 0.0019) and p-IAD SE rats (*q* = 4.752, *df* = 42, *p* = 0.0047). No significant difference was observed among EE offspring (Figure 1A).

### EE Rescues Recognition Memory in p-CAD Offspring

The results of the two-way ANOVA on the total time spent on the exploration of the two identical objects during the sample phase reveal a significant main effect of perinatal alcohol exposure (*F*_(2,42)_ = 11.21, *p* = 0.0001) and EE (*F*_(1,42)_ = 9.658, *p* = 0.0034). Tukey’s *post hoc* test shows that p-CAD SE offspring spent significantly less time exploring the objects than p-CTRL SE rats (*q* = 4.378, *df* = 42, *p* = 0.0382) and p-IAD SE rats (*q* = 4.797, *df* = 42, *p* = 0.0178). EE rats showed increased total exploration than SE offspring, with no significant pattern influence (Figure 1B).

Data analysis from familiar- and novel-object exploration during the test session included perinatal alcohol exposure and environmental rearing conditions as the between-subject factors and object as the within-subject factor. The results indicate a significant main effect of object (*F*_(1,42)_ = 62.673, *p* < 0.001), perinatal alcohol exposure (*F*_(2,42)_ = 8.501, *p* < 0.001), and rearing environment (*F*_(1,42)_ = 13.489, *p* < 0.001) and a significant interaction between perinatal alcohol exposure and rearing environment (*F*_(2,42)_ = 4.796, *p* = 0.013), object and perinatal alcohol exposure (*F*_(2,42)_ 13.506, *p* < 0.001), and object, perinatal alcohol exposure, and rearing environment (*F*_(2,42)_ = 14.367, *p* < 0.001). Pairwise comparisons with Bonferroni correction show that both p-CAD SE and p-IAD SE rats displayed a significant decrease in the exploration of the novel object, when compared to p-CTRL SE offspring (*p* < 0.001; *p* < 0.001; Figure 1C). In addition, while p-CTRL EE rats showed decreased exploration of the novel object, with respect to p-CTRL SE offspring (*p* < 0.001), p-CAD EE rats increased the exploration of the novel object with respect to their SE counterparts (*p* < 0.001; Figure 1C).

When RI% values from the test session were analyzed, two-way ANOVA, considering perinatal alcohol exposure and EE as statistical factors, showed a significant main effect of perinatal alcohol exposure (*F*_(2,42)_ = 6.812, *p* = 0.0027) and EE (*F*_(1,42)_ = 4.577, *p* = 0.0383) and a significant interaction (*F*_(2,42)_ = 6.348, *p* = 0.0039). In detail, Tukey’s *post hoc* test indicates a significant decrease in RI% of p-CAD SE- (*q* = 6.188, *df* = 42, *p* = 0.0010) and p-IAD SE rats (*q* = 4.835, *df* = 42, *p* = 0.0165), with respect to p-CTRL SE rats. EE rescued the RI% deficit in p-CAD rats (*q* = 5.581, *df* = 42, *p* = 0.0038), whereas no significant difference was observed between SE and EE p-IAD progeny (*q* = 1.121, *df* = 42, *p* = 0.9673; Figure 1D).

### EE Mitigates Spatial Learning and Memory Deficits in p-IAD Offspring

#### Spatial Learning in the Place Learning Task

**Figure 2:**
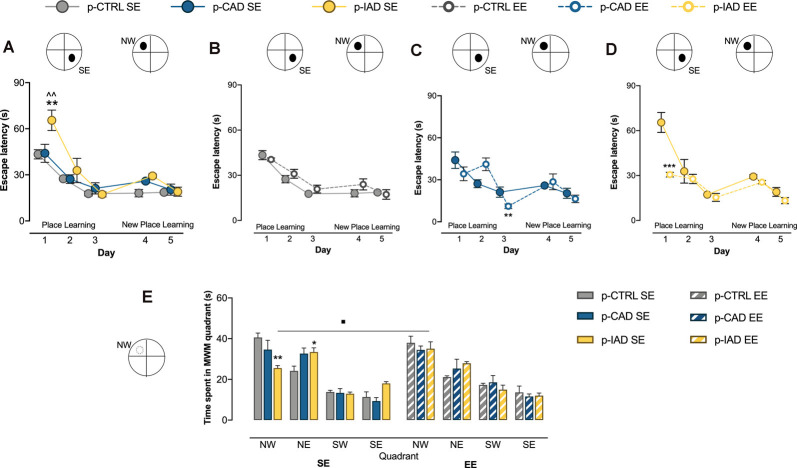
Effects of perinatal alcohol exposure and rearing conditions on spatial learning, cognitive flexibility, and reference memory. **(A)** p-IAD SE offspring showed a significant impairment in spatial learning (**p > 0.01 vs. p-CTRL SE; ^∧∧^*p* < 0.01 vs. p-CAD SE). **(B)** p-CTRL rats exposed to EE during adolescence did not differ from their SE counterparts. EE ameliorated the spatial learning performance in **(C)** p-CAD and **(D)** p-IAD offspring (**p < 0.01; ***p < 0.001 vs. respective SE counterparts). In addition, **(E)** p-IAD SE rats showed a reference memory deficit in the probe task, which was rescued by EE (*p < 0.05; **p < 0.01 vs. p-CTRL SE; ^▪^*p* < 0.05 vs. p-IAD SE). Each dot and each bar represent the mean ± SEM of *n* = 8 rats. p-CTRL, perinatal control; p-CAD, perinatal continuous alcohol drinking; p-IAD, perinatal intermittent alcohol drinking; SE, standard rearing environment; EE, enriched rearing environment. MWM, Morris water maze; NW, nord—west quadrant; NE, nord—east quadrant; SW, sud—west quadrant; SE, sud—east quadrant.

Data analysis performed on escape latency during the place learning task, when the offspring were trained to find the hidden platform over 3 days, considered perinatal alcohol exposure and rearing environment as the between-subject factors, and days as the repeated-measure factor. The results indicate a significant main effect of days (*F*_(2,84)_ = 80.256, *p* < 0.0001) and rearing environment (*F*_(1,42)_ = 5.636, *p* = 0.022) and a significant interaction between days and rearing environment (*F*_(2,84)_ = 12.319, *p* < 0.001), perinatal alcohol exposure and rearing environment (*F*_(2,42)_ = 5.048, *p* = 0.011), and among day, perinatal alcohol exposure, and rearing environment (*F*_(4,84)_ = 4.54, *p* = 0.002). Pairwise comparisons with Bonferroni correction show that p-IAD SE rats displayed increased escape latency with respect to p-CTRL SE (*p* = 0.003) and p-CAD SE (*p* = 0.004) offspring on day 1; in addition, p-CAD EE rats showed a significant decrease in escape latency with respect to p-CAD SE (*p* = 0.005) on day 3, whereas p-IAD EE offspring displayed a significantly decreased latency with respect to p-IAD SE rats (*p* < 0.001) on day 1 (Figures 2A–D).

#### Cognitive Flexibility in the New Place Learning Task

Statistical analysis on escape latency during the new place learning task, when the platform was moved to the NW quadrant, included perinatal alcohol exposure and rearing environment as the between-subject factors and days as the repeated-measure factor. The results reveal a significant main effect of days (*F*_(1,42)_ = 14.541, *p* < 0.001); perinatal alcohol exposure, rearing environment and their interactions displayed no significant effect (Figures 2A–D).

#### Spatial Reference Memory in the Probe Task

Data analysis performed on time spent in each of the MWM quadrants during the probe task included perinatal alcohol exposure and rearing environment as the between-subject factors and quadrant as the within-subject factor. The results show a significant main effect of quadrant (*F*_(2.250,94.499)_ = 85.652, *p* < 0.001) and a significant interaction between quadrant and perinatal alcohol exposure (*F*_(4.5,94.499)_ = 3.889, *p* = 0.004), quadrant and rearing environment (*F*_(2.25,94.499)_ = 3.051, *p* = 0.046), and perinatal alcohol exposure and rearing environment (*F*_(2,42)_ = 4.667, *p* = 0.015). Pairwise comparisons with Bonferroni correction indicate that p-IAD SE offspring spent significantly less time in the NW quadrant (*p* = 0.003), and longer time in the NE quadrant (*p* = 0.042) than p-CTRL SE rats. On the other hand, p-IAD EE rats spent increased time in the NW quadrant with respect to p-IAD SE rats (*p* = 0.028; Figure 2E).

### EE in Adolescence Blunts Long-Time Alcohol Vulnerability in p-CAD and p-IAD Offspring

#### Induction Period

**Figure 3:**
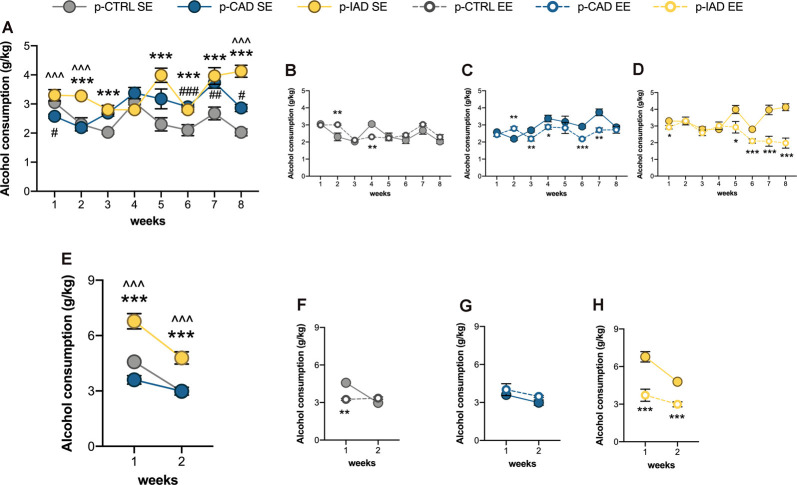
Effects of perinatal alcohol exposure and rearing conditions on alcohol consummatory behavior. **(A)** Apart from the first week of the two-bottle choice paradigm, both p-CAD- and p-IAD SE offspring showed increased alcohol intake with respect to p-CTRL SE rats; moreover, p-IAD SE rats displayed increased alcohol consumption with respect to p-CAD SE offspring (^#^*p* < 0.05; ^##^*p* < 0.01; ^###^*p* < 0.001 p-CAD SE vs. p-CTRL SE; ***p < 0.001 p-IAD SE vs. p-CTRL SE; ^∧∧∧^*p* < 0.001 p-IAD SE vs. p-CAD SE). EE exposure during adolescence. **(B)** Altered alcohol consumption in p-CTRL rats in **(C)** p-CAD rats and **(D)** decreased alcohol consumption p-IAD offspring (*p < 0.05; **p < 0.01; ***p < 0.001 vs. respective SE). **(E)** When offspring were assessed for alcohol deprivation effect during the relapse-like weeks, p-IAD-SE offspring showed higher alcohol intake with respect to p-CTRL SE and p-CAD SE rats; ***p < 0.001 vs. p-CTRL SE; ^∧∧∧^ vs. p-CAD SE). EE exposure during adolescence **(F)** decreased alcohol deprivation effect in p-CTRL offspring, **(G)** did not alter alcohol-related behavior in p-CAD rats, and **(H)** prevented alcohol deprivation effect in p-IAD offspring (**p < 0.01; ***p < 0.001 vs. respective SE). Each dot represents the mean ± SEM of *n* = 8 rats. p-CTRL, perinatal control; p-CAD, perinatal continuous alcohol drinking; p-IAD, perinatal intermittent alcohol drinking; SE, standard rearing environment; EE, enriched rearing environment.

Data analysis performed on mean alcohol intake along the 8 weeks of the two-bottle choice paradigm included perinatal alcohol exposure and rearing environment as the between-subject factors and weeks as the repeated-measure factor. The results show a significant main effect of weeks (*F*_(2.37,99.521)_ = 14.091, *p* < 0.001), rearing environment (*F*_(1,42)_ = 18.554, *p* < 0.001), and perinatal alcohol exposure (*F*_(2,42)_ = 13.807, *p* < 0.001) and a significant interaction between perinatal alcohol exposure and rearing environment (*F*_(2,42)_ = 10.225, *p* < 0.001), weeks and perinatal alcohol exposure (*F*_(4.739,99.521)_ = 6.668, *p* < 0.001), weeks and rearing environment (*F*_(2.37,99.521)_ = 9.576, *p* < 0.0001), and among weeks, perinatal alcohol exposure, and rearing environment (*F*_(4.739,99.521)_ = 7.559, *p* < 0.001). Pairwise comparisons with Bonferroni correction indicate that p-CAD SE offspring displayed decreased alcohol intake on week 1 (*p* = 0.012) and increased alcohol consumption on week 6 (*p* < 0.001), 7 (*p* = 0.003) and 8 (*p* = 0.011) with respect to p-CTRL SE rats. Moreover, p-IAD SE rats showed increased alcohol consumption on weeks 2 (*p* < 0.001), 3 (*p* < 0.001), 5 (*p* < 0.001), 6 (*p* < 0.001) 7 (*p* < 0.001), and 8 (*p* < 0.001) with respect to p-CTRL SE rats, along with increased alcohol intake on weeks 1 (*p* < 0.001), 2 (*p* < 0.001), and 8 (*p* < 0.001) with respect to p-CAD SE rats (Figure 3A).

EE modified alcohol consumption in p-CTRL rats, with a significant increase on week 2 (*p* = 0.001) and a decrease on week 4 (*p* = 0.001), when compared with the SE rearing condition (Figure 3B). Similarly, the enriched rearing environment increased alcohol intake in p-CAD offspring on week 2 (*p* = 0.007) and significantly decreased it afterward, on weeks 3 (*p* = 0.005), 4 (*p* = 0.016), 6 (*p* < 0.001), and 7 (*p* = 0.001) with respect to p-CAD SE rats (Figure 3C).

On the other hand, EE decreased alcohol intake in p-IAD progeny on weeks 1 (*p* = 0.021), 5 (*p* = 0.010), 6 (*p* < 0.001), 7 (*p* < 0.001), and 8 (*p* < 0.001) when compared with p-IAD SE counterparts (Figure 3D).

#### Relapse Period

The analysis of data from mean alcohol intake over the 2 weeks of the relapse paradigm included perinatal alcohol exposure and rearing environment as the between-subject factors and weeks as the repeated-measure factor. The results indicate a significant main effect of weeks (*F*_(1,42)_ = 76.57, *p* < 0.001), rearing environment (*F*_(1,42)_ = 17.112, *p* < 0.001), and perinatal alcohol exposure (*F*_(2,42)_ = 12.215, *p* < 0.001) and a significant interaction between perinatal alcohol exposure and rearing environment (*F*_(2,42)_ = 18.4513, *p* < 0.001), weeks and perinatal alcohol exposure (*F*_(2,42)_ = 5.371, *p* = 0.008), weeks and rearing environment (*F*_(1,42)_ = 24.567, *p* < 0.001), and among weeks, perinatal alcohol exposure, and rearing environment (*F*_(2,42)_ = 5.648, *p* = 0.007). Pairwise comparisons with Bonferroni correction indicate that p-IAD SE offspring showed higher alcohol intake than p-CTRL SE and p-CAD SE rats on week 1 (*p* < 0.001; *p* < 0.001) and week 2 (*p* < 0.001; *p* < 0.001; Figure 3E).

EE significantly decreased alcohol consumption in p-CTRL rats on week 1 (*p* = 0.008) with respect to their SE counterparts (Figure 3F) whereas no difference was observed between p-CAD SE and EE offspring (Figure 3G). On the other hand, p-IAD EE offspring displayed significantly lower alcohol intake on both week 1 and 2 (*p* < 0.001; *p* < 0.001), when compared to p-IAD SE rats (Figure 3H).

## Discussion

The present study aimed at evaluating the long-term consequences of maternal continuous- and binge-like intermittent alcohol drinking, from pre-conceptional time to lactation, on the adult male offspring’s cognitive behavioral readouts, including behavioral reactivity, declarative and spatial learning and memory, and alcohol vulnerability.

Moreover, we also exposed the offspring to an enriched rearing environment during adolescence, in order to evaluate whether sensorimotor stimulation and social interaction at that age could result in a rescue strategy able to mitigate or prevent perinatal alcohol-induced adverse effects.

In human studies, records on maternal blood alcohol levels are generally not available; however, estimates suggest that blood alcohol levels of over 200 mg/dl may be responsible for the severe FAS phenotype, while lower levels (80 mg/dl) may produce milder forms of FASD (Maier and West, 2001). In addition, high peaks of blood alcohol concentrations, rather than steady levels, as a result of both dose and pattern of alcohol exposure (i.e., binge-drinking vs. daily) during the brain developmental time-window, are associated with increased neurotoxicity (Ieraci and Herrera, 2007; Parnell et al., 2009).

In our experimental conditions, female rats were trained to voluntarily consume 20% alcohol in the drinking water prior to pregnancy (Patten et al., 2014) and consumed relevant amounts throughout pregnancy and lactation. In particular, CAD dams showed a mean daily alcohol consumption of 3.4 ± 0.4 g/kg during pregnancy, and 5.6 ± 0.6 g/kg during lactation, resulting in a daily low-to-moderate perinatal exposure for p-CAD offspring (Marquardt and Brigman, 2016). On the other hand, IAD dams engaged in a binge-like drinking pattern by every-other-day intermittent alcohol access to 20% alcohol, which resulted in mean alcohol consumption of 5.4 ± 0.6 g/kg during pregnancy and 8.5 ± 0.4 during the postpartum period. In particular, the IAD dams’ mean alcohol intake during the lactation period, measured after the first hour following alcohol presentation, is suggestive of an intermittent exposure to intoxicating blood alcohol concentrations for p-IAD offspring (>80 mg/dl, Loi et al., 2014). This evidence is particularly relevant since the intermittent pattern of exposure causes high peaks of blood alcohol concentrations during lactation in the rat dams, and this time window corresponds to the third developmental trimester in humans (Patten et al., 2014).

Our first data on the behavioral sequelae of perinatal alcohol exposure show pattern-related consequences on behavioral reactivity. In detail, p-CAD rats displayed a decrease in locomotor activity in the novel environment of the open field, with respect to p-CTRL and p-IAD rats, whereas p-IAD rats showed no alteration in total distance traveled, in comparison to p-CTRL. These results confirm early findings from this laboratory showing that perinatal long-term continuous exposure to alcohol decreased behavioral reactivity in the adolescent male offspring (Brancato et al., 2018). While moderate- and heavy-alcohol exposure during early-middle pregnancy either increased behavioral reactivity (Riley et al., 1993; Abel and Berman, 1994; Thomas et al., 2004; Kim et al., 2013) or did not affect locomotion (Dursun et al., 2006; Hellemans et al., 2010; Brady et al., 2012), the exposure to moderate alcohol concentration throughout gestation and the early postnatal period decreased locomotion in mice (Kleiber et al., 2011). Our data further suggest that the developmental effects of alcohol on locomotion and behavioral reactivity are affected not only by the dose and timing but also by the pattern of alcohol exposure.

In accordance with our first evidence, the analysis of the behavior in the sample phase of the novel object recognition test revealed that p-CAD rats showed a significant decrease in the exploration of the two identical objects when compared to both p-CTRL and p-IAD, while p-IAD rats explored the objects at the same extent as the control group did. Similarly, a decrease in exploration during the sample phase of the novel object recognition test was reported in Sardinian alcohol-preferring rats exposed to 3% alcohol from day 15 of gestation to day 7 after parturition (Tattoli et al., 2001) and interpreted as an altered responsiveness to situations requiring adaptation to novel environmental stimuli (Colombo et al., 1995).

On the other hand, the analysis of the test phase of the novel object recognition test suggested a deficit in declarative explicit memory, since both prenatal alcohol-exposed groups displayed a significant decrease in discrimination of the novel object: indeed, they spent the same time in the exploration of the familiar and the novel object, and this led to a significant decrease in the recognition index with respect to control offspring.

Preclinical findings have provided inconsistent evidence on the consequences of perinatal alcohol exposure on object discrimination, and the discrepancies are likely dependent on different times of exposure and blood concentrations.

In detail, alcohol exposure (dose range from 4.00 to 5.25 g/kg) during the developmental equivalent of the second and/or third trimesters in humans did not impair recognition memory in rats (Jablonski et al., 2013; Tattoli et al., 2001; MacIlvane et al., 2016), but when Sprague–Dawley female rats were given continuous unlimited access to alcohol from pre-conceptional period until weaning time, the offspring failed to discriminate the novel object in the object recognition test (Dandekar et al., 2019; Sanchez et al., 2019). Interestingly, maternal binge-like drinking during both gestation and lactation was reported to decrease recognition memory along with the expression of brain-derived neurotrophic factor (BDNF), the main neurotrophin involved in learning and memory (Montagud-Romero et al., 2019). Notably, even low levels of alcohol administered by oral gavage from GD 10–16 are able to exert a disruption in object recognition in the NOR, but not in object-place location; accordingly, this was associated with alterations in BDNF expression in the perirhinal cortex—a brain area which plays a crucial role in object discrimination-—rather than in the hippocampus, which is more involved in place location (Plescia et al., 2014c; Terasaki and Schwarz, 2017).

In our experimental conditions, perinatal alcohol exposure induced memory deficits regardless of the drinking pattern, suggesting an impairment in the regional circuitries underpinning declarative memory and that deserve attention from a translational point of view. However, it should not be overlooked that the recognition memory performance displayed by p-CAD rats could have been affected by their low behavioral reactivity and object exploration, rather than a pure deficit in declarative memory formation.

On the other hand, when offspring were tested for spatial learning and memory in the MWM, spatial navigation of p-CAD rats did not differ from control offspring, with no difference in spatial learning, in terms of latency to find the hidden platform over the 3 days of place learning, and in cognitive flexibility, along the 2 days of new place learning task. The evidence of no impairment in spatial reference memory supports the presence of regular spatial learning abilities in p-CAD progeny, since they searched the platform in the target quadrant during the probe trial, 24 h after the last new place learning session.

Taken together, our data are in line with previous reports demonstrating that chronic prenatal exposure to low-to-moderate doses of alcohol is sufficient to induce decreased behavioral reactivity in the open field (Kleiber et al., 2011) and declarative memory deficits in the novel object recognition test (Dandekar et al., 2019), together with a detrimental impact on the neuroimmune function of the perirhinal cortex (Terasaki and Schwarz, 2017). On the other hand, repeated low-dose prenatal alcohol exposure does not produce detrimental effects on pyramidal cells within the dorsal hippocampus or does not impair spatial learning and memory performance in the MWM (Cullen et al., 2014).

When interpreting these data, the stressful nature of the MWM task needs to be taken into account. The training in the MWM task increases the neuroendocrine stress response in rats, inducing high serum corticosterone concentrations that may affect the cognitive response in accordance with the positive role of glucocorticoids on learning and memory consolidation (Aguilar-Valles et al., 2005). It is reported that chronic alcohol exposure during pregnancy induces a reduction in ACTH basal levels while corticosterone secretion is not modified; besides, the exposure to some stressors induces an increase in corticosterone and CRH secretion, more than in controls (Lu et al., 2018). It is therefore reasonable to hypothesize that the stressful contingency of the MWM may boost the coping strategies of p-CAD progeny, by an “*ad hoc*” compensatory response of the HPA axis that makes p-CAD performance as “fair” as controls’ (Franks et al., 2020).

On the contrary, p-IAD offspring displayed a spatial learning impairment, in terms of increased latency to reach the hidden platform in the place learning test on day 1, compared to control offspring. In addition, p-IAD rats showed reference memory deficits, since they spent less time in the target quadrant in the probe trial, compared to p-CTRL groups. The impairment in spatial learning and reference memory in the water maze tasks is suggestive of hippocampal dysfunction likely resulting from the perinatal exposure to the binge-like alcohol drinking in the intermittent access. Indeed, despite some inconsistencies about alcohol-induced developmental effects on BDNF expression in the rat hippocampus (Feng et al., 2005; Ceccanti et al., 2012), binge-like alcohol exposure from one-to-third trimester-equivalent causes significant deficits in hippocampal and cortical neuroplasticity, resulting in alterations in dendritic arborization, adult, neurogenesis, neuroimmune activation in the hippocampus, and spatial learning impairment (Blanchard et al., 1987; Christie et al., 2005; An and Zhang, 2013; Harvey et al., 2019). Thus, due to the strong correlation between BDNF, hippocampal function and HPA axis reactivity, it is possible to interpret the current data on the basis of a pattern-specific effect exerted by the perinatal exposure to IAD on the stress axis response.

To our knowledge, the study by Wieczorek et al. (2015) is the only one focusing on HPA axis and behavioral sequelae of prenatal binge-like alcohol exposure. According to their findings, male mice exposed to an early binge-like dose of alcohol on gestational day 7 showed no difference in corticosterone levels with respect to controls, whereas they observed a blunted ACTH response to an acute stressor. Thus, it is reasonable to hypothesize that the exposure to intermittent alcohol drinking, which produces the cycling repetition of intoxications and withdrawals (Plescia et al., 2014a), when “brain growth spurt” and synaptogenesis occur (Patten et al., 2014), may impair spatial learning and memory in the MWM through a pronounced alteration in the neurodevelopmental programming of corticosteroid signaling in the hippocampus (Conrad et al., 1999).

The complex relationship between stress and alcohol is bidirectional, and the dysregulation of the stress response is a well-known risk factor for alcohol abuse vulnerability (Lee et al., 2018). The present data extend our previous findings and show that perinatal alcohol exposure is able to produce an alcohol-prone phenotype in adult rats in a pattern-related fashion. While p-CAD offspring increased their alcohol intake with respect to controls in the long-term, p-IAD rats showed a higher vulnerability to alcohol consummatory behavior starting from the first weeks of the two-bottle choice paradigm. In addition, while p-CAD-rats did not show higher consumption of alcohol after a week of deprivation with respect to control offspring, p-IAD progeny displayed a pronounced relapse behavior, when compared to both p-CAD and p-CTRL progenies. The alcohol deprivation effect is a reliable proxy of increased motivation to seek and consume alcohol, loss of control, and relapse (Spanagel and Hölter, 2000; Martin-Fardon and Weiss, 2013), and our data indicate that the perinatal exposure to a drinking pattern that promotes high peaks of blood alcohol level is discretely crucial in conferring a permanent vulnerability to alcohol abuse, whose occurrence can be detected since adolescence (Brancato et al., 2018).

This evidence supports clinical data showing that prenatally alcohol-exposed offspring display increased vulnerability to the rewarding properties of alcohol (Barbier et al., 2008) and risk for alcohol abuse and drug dependence later in life (Baer et al., 2003; Alati et al., 2006). This phenotype may result from morpho-functional alterations in the ventral tegmental area, such as decreased number of dopamine neurons and spontaneous action potentials, reduced size of their cell bodies, increased activated microglia (Shen et al., 1999; Aghaie et al., 2020), and persistent expression of immature excitatory synapses onto dopaminergic neurons (Wang et al., 2006). As far as the pattern-related behavioral abnormalities observed in this study concern, we could speculate that a dysregulation in the HPA axis may critically impact memory performance especially in the stressful setting of the MWM and may predispose to alcohol vulnerability (Brancato et al., 2014; Maniaci et al., 2015; Lee et al., 2018).

Notably, enriched rearing conditions ameliorated the behavioral performance of p-CAD rats in the novel object recognition test, likely remodeling p-CAD rats’ behavioral reactivity, decreasing emotionality and restoring those perceptive and attentive skills that make them able to overcome the cognitive impairment resulting from the perinatal continuous alcohol exposure. Accordingly, previous evidence showed that EE in early adulthood can recover cognitive impairment due to alcohol exposure during adolescence (Rico-Barrio et al., 2019). On the other hand, in our experimental conditions, declarative memory performance of p-IAD EE rats was not different from their SE counterparts’ one, suggesting that the abnormalities in declarative memory formation due to the perinatal intermittent exposure to alcohol are not rescued by the EE. Besides, the repeated exposure to environmental stimuli has been reported to decrease the incentive value of novelty (Cain et al., 2006; Garcia et al., 2017), suggesting that a lower interest in the novel object may explain its lower exploration by the EE offspring. Interestingly, our data show that EE mitigated the spatial learning and reference memory deficits induced by the perinatal intermittent alcohol paradigm. These findings are in agreement with previous reports, indicating that enriched environment attenuates hippocampal-dependent memory impairment induced by prenatal alcohol exposure, *via* an increase in hippocampal BDNF (Tipyasang et al., 2014; Di Liberto et al., 2017). The interpretation of the effects of EE upon alcohol vulnerability in the first weeks of the two-bottle-choice paradigm is not univocal since we observed mixed effects in the control offspring. In this regard, EE has been reported to promote the formation of conditioned place preference to alcohol in adolescent mice, likely recruiting the oxytocin signaling (Pautassi et al., 2017; Rae et al., 2018). However, the postweaning exposure to EE substantially rescued the increased vulnerability induced by perinatal alcohol exposure in p-CAD and p-IAD offspring. Indeed, EE decreased alcohol consumption in p-CAD and p-IAD rats, with respect to standard housing, during the last weeks of the self-administration paradigm. Thus, the increase in alcohol consumption, as time goes by, is a hallmark feature of early stages of the addiction cycle and represents a substantial risk factor predicting the development of alcohol addiction (Crabbe et al., 2011).

In addition, our data show that the enriched rearing environment decreases the deprivation effect after a week of forced abstinence, in p-IAD offspring and in p-CTRL rats. These observations are consistent with previous reports showing that exposure to enriched environmental conditions mitigates VTA dopamine neurons’ dysfunction due to perinatal alcohol exposure (Wang et al., 2018; Aghaie et al., 2020) and, overall, decreases the occurrence of an addictive-like phenotype (Galaj et al., 2020). Notably, the effect of the EE against the development of excessive alcohol intake seems to be protective when the exposure occurs during adolescence, while its protective role is limited when EE occurs during adulthood (Rodríguez-Ortega et al., 2018). To date, circumstantial evidence suggests that its protective effect against alcohol drinking is due to decreased CRH signaling in the amygdala and its downstream target (Sztainberg et al., 2010). Whether CRH abnormalities may be the *primum movens* for the occurrence of the dysfunctional phenotype consequent to perinatal alcohol exposure observed in this study, and at what extent alteration in maternal care can contribute to alcohol developmental effects, are interesting questions to address in further cross-fostering experiments. Moreover, studies including female offspring are needed to explore sex differences in the developmental effects of alcohol, and their underlying mechanisms. Overall, subsequent developmental periods, such as adolescence, provide a window of opportunity for inducing positive experience-based neuroplasticity in brain regions critical for emotional regulation, cognitive functions, and reward sensitivity, which allow curtailing the lifetime consequences of developmental alcohol exposure.

## Data Availability Statement

The raw data supporting the conclusions of this article will be made available by the authors, without undue reservation.

## Ethics Statement

The animal study was reviewed and approved by Committee for the Protection and Use of Animals of the University of Palermo.

## Author Contributions

AB: experimental procedures and data analysis and contribution to writing. VC and GL: experimental procedures and contribution to writing. CC: experimental design, data interpretation, and writing. All authors contributed to the article and approved the submitted version.

## Conflict of Interest

The authors declare that the research was conducted in the absence of any commercial or financial relationships that could be construed as a potential conflict of interest.
